# Efficacy and Safety of Ivonescimab in the Treatment of Advanced Non-small Cell Lung Cancer (NSCLC): A Systematic Review

**DOI:** 10.7759/cureus.77381

**Published:** 2025-01-13

**Authors:** Alzahra'a Al Matairi, Bara M Hammadeh, Abdullah Yousef Aldalati, Fares A Qtaishat, Abdulqadir J Nashwan, Abdulla Alzibdeh

**Affiliations:** 1 Medical Oncology, Faculty of Medicine, University of Jordan, Amman, JOR; 2 Medical Oncology, Faculty of Medicine, Al-Balqa’ Applied University, Amman, JOR; 3 Medical Oncology, Faculty of Medicine, Jordan University of Science and Technology, Irbid, JOR; 4 Public Health, College of Health Sciences, QU Health, Qatar University, Doha, QAT; 5 Radiation Oncology, King Hussein Cancer Center, Amman, JOR

**Keywords:** adverse events (aes), anti-pd-1, ivonescimab, nsclc, vegf

## Abstract

This systematic review critically evaluates the safety and efficacy of the novel drug Ivonescimab in the treatment of advanced and metastatic non-small cell lung cancer (NSCLC). Ivonescimab showed promising antitumor activity and improved clinical outcomes, particularly in patients with higher PD-L1 expression levels and those receiving second-line therapy. The findings suggest its potential to overcome resistance to PD-1/PD-L1 inhibitors while offering a manageable safety profile. Common adverse events were observed, highlighting the need for further research to refine dosing strategies and optimize patient selection. Future studies should focus on long-term outcomes and real-world applications to better establish the role of Ivonescimab in NSCLC management.

## Introduction and background

Lung cancer is one of the most prevalent malignancies and has a significant worldwide fatality rate [1,2]. Non-small cell lung cancer (NSCLC) is the most common type of lung cancer worldwide, accounting for approximately 85% of all lung cancer cases [3,4]. It is a heterogeneous group of tumors, classified according to their histological appearance into three main subtypes: adenocarcinoma, squamous cell carcinoma, and large cell carcinoma. Among these, adenocarcinoma is the most prevalent, especially among nonsmokers [4,5].

NSCLC is often diagnosed at an advanced or metastatic stage [6], where treatment options are limited, and the prognosis remains poor. Almost 30-40% of patients are diagnosed with de novo metastatic disease, and the majority are diagnosed with advanced-stage disease (stage III or IV). The 5-year relative survival rate is 26% in all stages and only 8% in cases with distant metastases [7].

The treatment options for NSCLC have undergone several changes over the past few decades. Initially, patients mostly received chemotherapy, which yielded few benefits. However, as research has advanced, treatment approaches have expanded to include a broader range of therapies for various NSCLC subtypes, such as squamous cell carcinoma and large cell carcinoma, in addition to adenocarcinoma. These advancements have been pivotal in improving patient outcomes across different NSCLC subtypes [8-10]. Recent advances have led to the emergence of newer and more efficient treatments such as immunotherapy and targeted therapy [11].

The introduction of immune checkpoint inhibitors (ICIs), including those targeting the PD-1/PD-L1 axis, has revolutionized NSCLC treatment, offering improved survival compared with traditional chemotherapy [12-14]. However, resistance to ICIs is a substantial therapeutic challenge, as their efficacy decreases with time and disease progression develops [15]. These limitations highlight the need for novel therapeutic approaches that may overcome resistance and enhance long-term outcomes for NSCLC patients with NSCLC.

Ivonescimab is a bispecific antibody that targets both PD-1/PD-L1 and vascular endothelial growth factor (VEGF). It is considered a novel treatment due to its dual mechanism of action. By simultaneously inhibiting the PD-1/PD-L1 axis and VEGF, Ivonescimab aims to enhance anti-tumor immunity while also addressing the tumor vasculature. This dual-targeting approach offers the potential to overcome resistance mechanisms commonly encountered with single-target therapies, providing a more comprehensive therapeutic strategy for patients with NSCLC [16]. VEGF is a major factor in angiogenesis, which promotes the development of new blood vessels and hence aids tumor growth [17]. Ivonescimab inhibits both PD-1/PD-L1-mediated immune evasion and VEGF-driven angiogenesis, providing a dual mechanism of action that improves therapeutic effectiveness in NSCLC. Early clinical studies have shown promising results, with Ivonescimab showing strong anticancer efficacy and manageable safety profile in patients with NSCLC [18,19]. This systematic review aimed to critically evaluate the efficacy and safety of Ivonescimab in treating advanced and metastatic NSCLC by synthesizing data from available clinical studies.

## Review

Materials and methods

This systematic review followed the Preferred Reporting Items for Systematic Reviews and Meta-Analyses (PRISMA) guidelines [20]. All stages adhered to the Cochrane Handbook for Systematic Reviews of Interventions [21]. The review was registered with PROSPERO in October 2024 with registration number CRD42024600405.

Search Strategy and Eligibility

We systematically searched the PubMed, Scopus, Science Direct, and Cochrane Library databases from their inception to October 1, 2024 (Appendices). The search terms included (“Ivonescimab” OR “AK112” AND “NSCLC” OR “Non-small cell lung cancer”). In addition, we reviewed the reference lists of the selected articles to ensure a comprehensive search. The search information for each database can be found in the supplementary material S1. Two reviewers performed a literature search and selected studies based on the following criteria: patients aged 18 years or older with newly diagnosed advanced or metastatic NSCLC confirmed by pathology receiving Ivonescimab alone or in combination with chemotherapy compared with patients receiving placebo of chemotherapy alone. The exclusion criteria were as follows: 1. Studies without reported clinical outcomes related to efficacy or safety; 2. Reviews; 3. Conference abstracts; 4. Case reports or case series; 5. Studies that focused solely on the molecular mechanisms of the drug.

 *Study Selection and Data Extraction*

Two authors independently screened the titles and abstracts of all identified articles based on predetermined inclusion criteria. Full-text reviews were independently conducted for potentially relevant studies to confirm their eligibility. The final selection of studies was determined by consensus among all authors. Data extraction was systematically performed by the same reviewers using a standardized form [21]. Extracted information includes baseline characteristics (histologic features, Eastern Cooperative Oncology Group (ECOG) performance, PD-L1 tumor proportion score), efficacy outcomes (overall response rate, overall survival, progression-free survival), and safety endpoints (adverse events).

Quality of Studies and Risk of Bias Assessment

We used the risk of bias in the non-randomized studies of interventions (ROBINS-I) tool to evaluate observational cohort studies in six domains [22]. We also used version 2 of the Cochrane risk-of-bias tool for randomized trials (ROB 2) from the Cochrane Handbook of Systematic Reviews of Interventions 6.3 for randomized controlled trials (RCT) in five domains [21,23]. Each domain was answered, and an overall judgment was provided for the prosecution. The risk of bias was categorized as low, concerning, or high.

Results

Search Result

**Figure 1:**
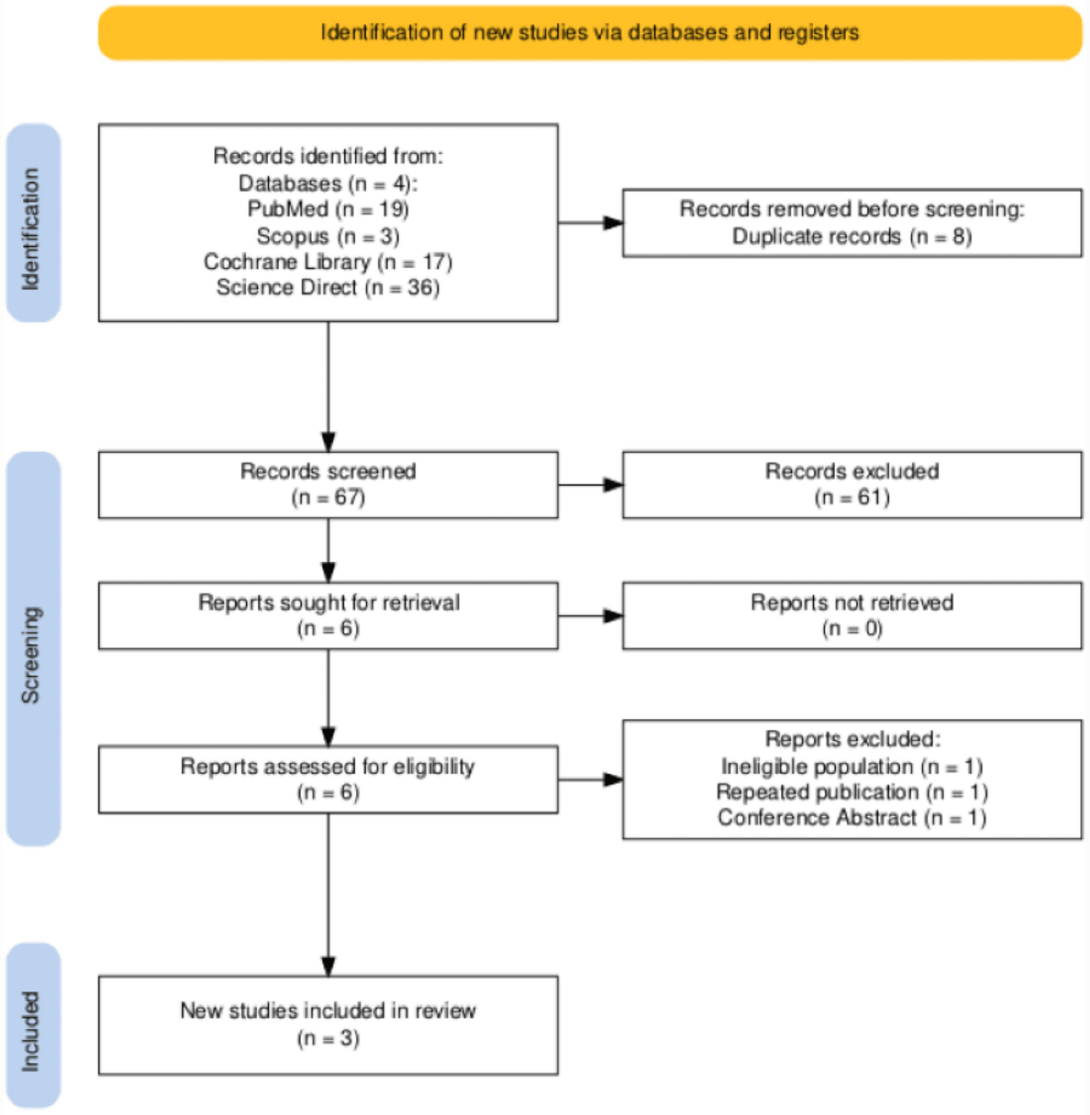
PRISMA flow diagram of the selection process PRISMA: Preferred Reporting Items for Systematic Reviews and Meta-Analyses

The initial search strategy yielded 75 studies. After removing duplicates, 67 studies remained. Screening of titles and abstracts resulted in the selection of six articles for full-text review. Ultimately, three manuscripts met the inclusion criteria and were included in this review [24-26]. The study selection process is illustrated in the PRISMA flowchart in Figure 1.

Summary of Included Results

We reviewed 513 adults with non-small cell lung cancer and 161 controls aged 18-75 years. Overall survival (OS), progression-free survival (PFS), objective response rate (ORR), ECOG performance, disease stage, PD-L1 tumor proportion score, and adverse events were extracted.

Quality Assessment

We conducted a comprehensive quality assessment on all trials presented as full manuscripts to evaluate the inherent risk of bias. In total, we included three studies in this review. Of these, one randomized controlled trial and two non-randomized observational cohort studies (phase I and phase II studies). None of the studies were excluded based on the risk of bias, as no evidence suggested that the risk of bias significantly affected the effect estimates.

Regarding the RCT, while it was judged to have a low risk of bias, some concerns were noted in specific areas such as allocation concealment and blinding. These concerns were not substantial enough to exclude the study but were acknowledged to ensure transparency and completeness in the quality assessment. The two observational studies were also assessed for bias, but they were considered to have a low risk of bias as well.

Study Characteristics

Of the three included studies, one was a randomized clinical trial while the other two were phase I and II non-randomized trials. In one study, participants were divided into three cohorts: the first cohort received first-line AK112 (Ivonescimab) with platinum-based chemotherapy, the second cohort included patients with EGFR-sensitive mutations who had failed prior targeted therapy, and the third cohort consisted of patients who had failed prior systemic platinum-based chemotherapy and PD-1/L1 inhibitor treatment [26]. Another study evaluated the effects of various dosing regimens, including 10 mg/kg Q3W, 20 mg/kg Q2W, 20 mg/kg Q3W, and 30 mg/kg Q3W [24]. The final study compared Ivonescimab plus chemotherapy with placebo plus chemotherapy [26]. The baseline disease stages were III and IV, and the ECOG performance status was either 0 or 1. The basic characteristics of the included studies are shown in Table 1.

**Table 1:** Characteristics of included studies

Author	Year	Country	Study design	Treatment	No. of patients	Male (%)	Median age (Range)	Median follow-up (months)
Fang et al. (23)	2024	China	Randomized Phase 3 Trial	Ivonescimab plus chemotherapy	161	77 (47.8)	59.6 (32.3-74.9)	7.9
				Chemotherapy	161	79 (49.1)	59.4 (36.2-74.2)	
Wang et al. (17)	2023	China	Cohort Phase 1	10 mg/kg Q3W	30	23 (76.7)	64 (48-74)	10.4
				20 mg/kg Q2W	29	26 (89.7)	68.0 (51–74)	
				20 mg/kg Q3W	29	25 (86.2)	65.0 (53–75)	
				30 mg/kg Q3W	20	17 (85.0)	66.0 (51–73)	
Zhao et al. (22)	2023	China	Cohort Phase 2	AK112 + carboplatin + pemetrexed for non-squamous or paclitaxel for squamous	44	105 (77.8)	57.6 (44.3–73.0)	12.7
				AK112 + carboplatin + pemetrexed	19	6 (31.6)	60.2 (34.7–64.9)	
				AK112 + docetaxel	20	16 (80.0)	60.0 (31.6–73.4)	

Outcomes

Effect of Ivonescimab on advanced NSCLC:* *Regarding Zhao et al., participants receiving 20 mg/kg of first-line AK112 (Ivonescimab) with platinum-based chemotherapy had a higher, though non-significant, ORR compared to those receiving 10 mg/kg in the first cohort [26]. The ORR was 68.4% in the second cohort, comprising patients with EGFR-sensitive mutations who had failed prior targeted therapy, and 40% in the third cohort, comprising patients who had failed prior systemic platinum-based chemotherapy and PD-1/L1 inhibitor treatment [26]. Across all cohorts, the ORR was higher in patients with PD-L1 expression levels of 1%-49% and ≥50% than in those with negative PD-L1 expression [26]. Additionally, the ORR in the Ivonescimab group was significantly higher than that in the placebo group [25]. Patients with squamous NSCLC have a 13.1% higher ORR than those with non-squamous NSCLC [24,26]. Higher doses (20 and 30 mg/kg) and increased dosing frequencies (Q3W compared to Q2W) were associated with ORRs ranging from 33.3% to 75% [24]. Patients with a tumor proportion score (TPS) of ≥50% had higher ORRs than those with TPS scores of 1%-49% [24]. In second-line therapy, patients with TPS ≥1% have higher ORRs than those with TPS <1% [24]. Furthermore, 39.8% of patients achieved ORR after receiving at least one dose of Ivonescimab [24].

Zhao et al. reported that the median PFS varied between cohorts: the first cohort did not reach a median PFS value, the second cohort had a median PFS of 8.5 months with 11 disease progression events, and the third cohort had a median PFS of 7.5 months [26]. PFS decreased with longer follow-up durations, ranging from 3 to 12 months, with the highest PFS reported in cohort 1 [24,26]. The intervention group consistently had higher PFS rates at 3, 6, and 12 months than the control group [25]. Ivonescimab improved PFS in multiple subgroups, including patients who progressed to EGFR-TKI therapy, Ivonescimab plus chemotherapy versus chemotherapy alone, and patients with EGFR exon 19 deletion and T790M mutations, with the latter having a lower hazard ratio (HR) [25]. The median PFS was longer in patients with non-squamous NSCLC than in those with squamous NSCLC [24], and the six-month PFS was higher than in nine-month PFS with patients receiving ≥20 mg/kg [24].

Regarding the disease control rate (DCR), Zhao et al. reported percentages ranging from 70% to 93%, with cohort 1 showing the highest DCR [26]. Patients receiving Ivonescimab had a significantly higher DCR than those receiving a placebo [25]. The duration of response (DOR) was also longer in the Ivonescimab group [25]. One study noted a decrease in tumor volume in all but one patient in the 10 mg/kg group during treatment, with volume changes over time indicating a benefit from Ivonescimab treatment regardless of histological characteristics or dosage levels [26]. Tumor burden reduction and durable responses were observed in most patients, regardless of PD-L1 TPS status or prior systemic therapy [24].

Adverse Events

In two studies, almost all participants in the Ivonescimab group experienced treatment-emergent adverse events (TEAEs) at rates of 100% [26] and 99.4% [25], respectively. In contrast, the placebo group had a slightly lower TEAE rate (97.5%) [25]. Eleven patients discontinued treatment because of TEAEs [25,26], and one patient discontinued treatment because of a treatment-related serious adverse event (TRSAE) [24]. Serious adverse events (SAEs) were reported in 41% of patients in the Ivonescimab group compared with 25.2% in the control group [25].

The incidence of grade ≥3 treatment-related adverse events (TRAEs) was higher in patients with squamous NSCLC compared to those with non-squamous NSCLC. However, all-grade bleeding-related adverse events are more frequent in patients with non-squamous NSCLC [24]. A total of 21 patients in the Ivonescimab group experienced TEAEs leading to death across studies [24-26], with all causes of death attributed solely to TRAEs [26]. TEAEs leading to either discontinuation or death were more common in the Ivonescimab group than those in the placebo group.

Additionally, a lower percentage of grade ≥3 TRAEs and TRSAEs was observed with Q3W dosing than with Q2W dosing [24]. Immune-related adverse events (irAEs) were reported in 77 patients treated with Ivonescimab, with 16 patients experiencing grade 3 or higher irAEs. The incidence of irAEs was higher in the Ivonescimab group than in the control group [24-26]. VEGF-related adverse events occurred in 110 patients in the Ivonescimab group, with a higher incidence compared to the control group [24,25].

Discussion

To our knowledge, this is the first systematic review to provide a comprehensive review of the efficacy and safety of Ivonescimab in the treatment of patients with NSCLC. Ivonescimab demonstrated promising results and was generally well-tolerated. The combination of Ivonescimab and chemotherapy resulted in better ORRs and PFS rates at 3, 6, and 12 months. Furthermore, Ivonescimab was significantly effective in reducing the tumor burden, with nearly all patients experiencing measurable decreases in tumor volume. The incidence of AEs was generally higher in the Ivonescimab group.

This review synthesized findings from three key trials on Ivonescimab in advanced NSCLC. The HARMONi trial, a Phase 3 randomized study, was the first trial to demonstrate a notable clinical benefit of Ivonescimab plus chemotherapy in patients with EGFR-TKI-resistant NSCLC [25]. Ivonescimab significantly improved progression-free survival compared to chemotherapy alone (HR, 0.46; P < .001), with higher PFS rates at 3, 6, and 9 months, marking a significant advancement in treatment for this patient population. The Phase 1b study conducted by Wang L et al. represented the first-in-human evaluation of Ivonescimab monotherapy and demonstrated a tolerable safety profile [24]. The study revealed dose-dependent antitumor activity, particularly in PD-L1-positive patients treated with doses ≥20 mg/kg in the first line setting. This was the first study to explore a bispecific antibody targeting both PD-1 and VEGF for NSCLC treatment without chemotherapy. Lastly, the Phase 2 trial conducted by Zhao Y was the first to evaluate the efficacy and safety of Ivonescimab in combination with chemotherapy for metastatic NSCLC [26]. The study highlighted the broad applicability of Ivonescimab across multiple therapy lines, including first-line treatment for advanced NSCLC without driver mutations and treatment in patients with EGFR mutations or prior PD-1/L1 inhibitor failures. It also confirmed that AK112 was generally well-tolerated across all patient groups.

ORRs ranged from 33.3% to 75% across the included studies. Patients with PD-L1 expression levels of 1%-49% or ≥50% achieved higher ORRs than those with negative PD-L1 expression. Similarly, patients with TPS ≥50% consistently exhibited better response rates than those with TPS scores of 1%-49%. This was expected, as Ivonescimab targets both PD-1 and VEGF. Additionally, patients with squamous NSCLC demonstrated a 13.1% higher ORR than those with nonsquamous NSCLC. Although this could suggest that squamous NSCLC responds better to Ivonescimab, the reason for this is not fully understood [26]. Further research is required to compare the effects of Ivonescimab on the different histological patterns.

Improvements in PFS were observed across all included studies and various dose regimens. Ivonescimab consistently showed better PFS than placebo or chemotherapy alone at 3, 6, and 12 months. The intervention group showed a median PFS of 7.1 months, whereas the control group showed 4.8 and a median PFS of 4.8 months. The 6- and 9-month PFS rates ranged from 55.4% to 64.1% and from 37.9% to 52.2%, respectively. This consistently favorable result highlights its potential as an effective treatment option for patients with advanced NSCLC. In contrast to the ORR, the median PFS was longer in patients with non-squamous NSCLC than in those with squamous NSCLC. This difference in results highlights the complexity of treatment responses across different histological subtypes and further increases the need for further research.

In a study conducted by Wang et al., participants receiving 10 mg/kg Q3W had an ORR of 33.3%, which improved to 60.0% in the 20 mg/kg Q3W cohort and further to 75.0% in the 30 mg/kg Q3W cohort [24]. The study by Zhao et al. reported smaller differences in ORRs, as participants receiving 10 mg/kg achieved an ORR of 52.6%, while those receiving 20 mg/kg had a slightly higher ORR of 54.2% [26]. These results indicated a trend toward better efficacy with higher doses within the same dosing interval.

An ongoing trial, the HARMONi-2 phase 3 trial, has brought forward promising evidence that could redefine the first-line treatment landscape for advanced NSCLC [18]. Presented by Dr. Caicun Zhou at the 2024 World Conference on Lung Cancer, this trial compared Ivonescimab with pembrolizumab in patients with stage IIIB to IV advanced NSCLC. Ivonescimab demonstrated a striking PFS advantage, with a median PFS of 11.14 months compared to 5.82 months for pembrolizumab (HR, 0.51; P < .0001). The benefits were consistent across subgroups, including varying PD-L1 expression levels and histologies (squamous and nonsquamous NSCLC). While overall survival data remain immature, Ivonescimab also showed higher overall response and disease control rates, alongside a manageable safety profile similar to pembrolizumab.

Several clinical trials have demonstrated the advantages of integrating ICIs into the treatment plan. The Mpower-150 trial evaluated atezolizumab in combination with bevacizumab and chemotherapy for advanced NSCLC [27]. This regimen achieved an ORR of 63.5% and a PFS of 8.3 months. The PFS rates at 6 and 12 months were 66.9% and 36.5%, respectively. Dual immunotherapy combinations targeting PD-1 and CTLA-4 have been investigated in the CheckMate 9LA and POSEIDON trials [28,29]. In the CheckMate 9LA trial, nivolumab and ipilimumab combined with chemotherapy achieved an ORR of 38.2%, with a median PFS of 6.7 months and a 12-month PFS rate of 33% [28]. Similarly, the POSEIDON trial evaluated tremelimumab and durvalumab with chemotherapy, resulting in a slightly higher ORR of 38.8%, although the median PFS was slightly shorter at 6.2 months and the 12-month PFS rate was 26.6% [29]. Based on these results, Ivonescimab demonstrated slightly better efficacy in terms of both ORR and PFS.

AEs are a significant consideration in immune-targeting therapies, particularly those targeting ICIs such as anti-PD-1, anti-PD-L1, and anti-CTLA-4 agents [30]. Ivonescimab plus chemotherapy had an acceptable AE rate compared with other immune-targeting therapies. The rate of grade 3-4 TRAEs observed with this regimen was substantially lower than that reported in other trials (Mpower-150, CheckMate 9LA, POSEIDON trials), with 26.5% compared to 58.5%, 47%, and 51.8%, respectively [27-29]. The irAEs associated with Ivonescimab were typically low-grade and manageable. 

Limitations

The limitations of this study must be acknowledged. The sample sizes of the included studies were relatively small. In addition, we were unable to conduct a meta-analysis because the data were heterogeneous and could not be pooled together. Although Ivonescimab was especially effective in certain subtypes, more RCTs with larger sample sizes for each subgroup are required to draw a definitive conclusion.

## Conclusions

This systematic review concludes that Ivonescimab has shown satisfactory efficacy and relatively safe outcomes in treating patients with advanced NSCLC. Ivonescimab, especially when taken with chemotherapy, had shown higher ORRs and PFS compared to chemotherapy or placebo treatment and therefore might provide a treatment for patients with advanced or chemo-refractory disease. The results indicated that higher PD-L1 scores for expression and TPS are associated with improved outcomes, which implies that indicators can be used to identify suitable candidates for therapy. AEs were common but were mainly mild to moderate in severity and presented at a frequency similar to or lower than those described for other immune-targeted therapies. However, the small sample sizes and heterogeneity of the included studies highlight the need for larger, well-designed RCTs to confirm these findings and explore the potential benefits across different histological subtypes and dosing regimens.

## Appendices

Detailed search strategies

This document provides detailed information on the search strategies used in our systematic review. We systematically searched the PubMed, Scopus, Science Direct, and Cochrane Library databases from their inception to October 1, 2024. The search terms included ('Ivonescimab' OR 'AK112' AND 'NSCLC' OR 'Non-small cell lung cancer'). We also reviewed the reference lists of the selected articles to ensure a comprehensive search.

Search results summary

**Table 2:** Search results summary

Category	Count
PubMed	19
Scopus	3
Science Direct	36
Cochrane	17
Total before duplicate removal	75
Total duplicates removed	8
Total after duplicate removal	67
Total after title and abstract screening	6
Total after full-text screening	3
Total included in the review	3

Database-specific search strategies

1. PubMed

Search Query: ("Non-Small Cell Lung Cancer" OR NSCLC OR "Non-Squamous Non-Small Cell Lung Cancer" OR "Squamous Non-Small Cell Lung Cancer") AND (Ivonescimab OR "PD-1/VEGF bispecific antibody" OR AK112 OR "anti-PD-1 VEGF inhibitor")

Filters Applied: None.

Results Retrieved: 19

2. Scopus

Search Query: TITLE-ABS-KEY ("Non-Small Cell Lung Cancer" OR NSCLC OR "Non-Squamous Non-Small Cell Lung Cancer" OR "Squamous Non-Small Cell Lung Cancer") AND TITLE-ABS- KEY (Ivonescimab OR "PD-1/VEGF bispecific antibody" OR AK112 OR "anti-PD-1 VEGF inhibitor")

Filters Applied: None.

Results Retrieved: 3

3. Science Direct

Search Query: ("Non-Small Cell Lung Cancer" OR NSCLC OR "Non-Squamous Non-Small Cell Lung Cancer" OR "Squamous Non-Small Cell Lung Cancer")  AND  (Ivonescimab OR "PD-1/VEGF bispecific antibody" OR AK112 OR "anti-PD-1 VEGF inhibitor")

Search Fields: Article Title, Abstract, and Keywords.

Filters Applied: None.

Results Retrieved: 36

4. Cochrane Library

Search Query: ('Ivonescimab' OR 'AK112') AND ('NSCLC' OR 'Non-small cell lung cancer')

Filters Applied: None.

Results Retrieved: 17

5. Manual Reference Checking

We manually reviewed the reference lists of the included studies to ensure no relevant studies were omitted.
